# Why do people fail to turn good intentions into action? The role of executive control processes in the translation of healthy eating intentions into action in young Scottish adults

**DOI:** 10.1186/1471-2458-8-123

**Published:** 2008-04-18

**Authors:** Julia L Allan, Marie Johnston, Neil Campbell

**Affiliations:** 1 School of Psychology, University of Aberdeen, Aberdeen, UK; 2 Health Services Research Unit, University of Aberdeen, Aberdeen, UK; 3 Department of General Practice and Primary Care, University of Aberdeen, Aberdeen, UK

## Abstract

**Background:**

Despite the significant health benefits associated with eating healthily, diet is extremely difficult to change, with the majority of people who intend to eat more healthily failing to do so. Recent evidence has suggested that the ability to turn intentions into actions may be related to individual differences in one facet of executive control – cognitive inhibition (i.e. the ability to inhibit irrelevant information and suppress prepotent responses). The present study investigates the role of this and other executive processes (inhibition, task switching, planning and cognitive flexibility) in the translation of dietary intentions into action. In addition, as the literature suggests that weak executive control may be associated with hyper-responsivity to cues to action, the role of executive processes in susceptibility to environmental food cues and responses to If-Then plans designed to cue intended behaviour are investigated.

**Methods:**

Future intentions about consumption of fruits and vegetables and snack foods will be measured in a sample of young adults. Actual consumption of the target foods will be recorded with computerised diaries over a subsequent 3-day period. Performance on a battery of established executive control tasks (Go-NoGo, Tower task, Verbal Fluency task and Trail-Making) will be used to predict the discrepancy between intended and actual dietary behaviour. In addition, executive control scores will be used to predict reported susceptibility to environmental food cues and benefit derived from the use of 'If-Then plans' designed to cue intended behaviour.

**Discussion:**

Our findings will add to understanding about the role of executive control in translating intentions into actions and may demonstrate potential for future public health interventions. If participants with weak executive control are found to be less likely to eat as they intend than those with strong executive control, then interventions that reduce the load on these executive processes may increase chances of successful intention-behaviour translation. If those with weak executive control are found to be more responsive to cues to action they may also benefit more from the use of If-Then plans designed to cue intended behaviour.

## Background

In common with all developed countries, the Scottish diet is high in fat, salt and sugar and low in fruits and vegetables [1]. As this pattern of dietary behaviours is associated with rising levels of obesity and associated chronic diseases, diet is an important target for change in government strategies aimed at improving public health [2]. Although many factors aside from diet are known to contribute to rising levels of obesity, research from the United States suggests that poor diet in combination with physical inactivity is close to overtaking smoking as the leading cause of premature death [3].

While providing the public with information about healthy eating can increase intentions towards the adoption of healthier diets, it is ultimately down to the individual whether good intentions are translated into action. As highlighted in the Scottish Diet Action Plan [4], the majority of the Scottish public "know what makes a healthy diet and what does not". The problem instead is "the widespread failure to act on this knowledge" (p2).

Psychological research has established that only 30–40% of lifestyle intentions are successfully realised [5,6], leaving a considerable 'intention-behaviour gap'. If factors determining the success or failure of intentions about lifestyle intentions can be identified, it may be possible to facilitate positive behaviour change on a large scale, across a range of key behaviours.

One possible determinant of successful intention-behaviour translation is efficient cognitive control, i.e. whether people can organise and direct their thinking towards enacting intended behaviour. Cognitive or 'executive' control processes are responsible for instigating and controlling task-directed thoughts and behaviours, and include selective attention, planning, cognitive inhibition (ignoring irrelevant information/suppressing habitual responses that run counter to aims), task-set reconfiguration (shifting your way of thinking to suit your aims), and flexibility of thought [7]. In theory, all of the aforementioned processes are prerequisites for the successful execution of behaviour, and consequently differences in executive strength may explain differences in ability to turn intentions into action. Recent evidence has shown at least one executive process (cognitive inhibition) does indeed predict variance in diet over a 7 day period over and above the variance explained by an individual's intentions [8]. Importantly, those scoring poorest on the executive test (i.e. with the weakest executive control) showed the largest discrepancy between intended and actual behaviour. The latter research was based on Temporal Self-Regulation Theory [9], which makes the further prediction that the likelihood of performing a behaviour is a function of both an individual's self-regulatory capacity (i.e. executive control), and the presence or absence of cues to that behaviour in the environment. That is, if cues to eating were present in the environment, the likelihood of eating would increase, but this increase would be greatest in those with poor executive control, as they would be less able than those with stronger control to inhibit task irrelevant information.

If those with weak executive control are more likely to behave in response to cues, then in addition to potentially being more susceptible to advertising, they may also be more likely to benefit from interventions designed to cue an individual into performing the target behaviour. 'Implementation intentions' or If-Then plans [10] are plans which ask individuals to specify when and where they intend to carry out a behaviour, and despite their simplicity have been shown to increase rates of many different health behaviours such as breast self-examination [11], adherence to dietary supplement regimes [12], exercise [13] and eating more fruits and vegetables [14]. Implementation intentions are thought to work because after specifying the If-Then contingencies associated with behaviour, encountering the If component (e.g. it gets to the time specified in the plan), should elicit the behaviour relatively automatically, without much need for effortful executive control. If this is the case, then those with weak executive control should benefit more than others from the formation of implementation intentions.

The present project aims to expand on recent empirical work [8] by looking at the role of multiple executive processes, specifically inhibition, task-switching, cognitive flexibility and planning, in the ability to perform intended behaviour. In addition, the prediction that those with weak executive control will be more likely to eat in response to external food cues than those with strong executive control will be tested, and the benefit of implementation intentions in those with weak and strong executive control will be compared. Finally, performance on the executive control tasks will be compared to scores on self-report measures of executive control, the Dysexecutive Questionnaire [15] and the Cognitive Failures Questionnaire [16], to establish for a future study which aspects of executive control self-report measures detect.

## Methods/Design

### Aims

(1) To establish the extent to which 4 cognitive control processes (cognitive inhibition, cognitive flexibility, planning, and task-switching) determine the success or failure of intentions about the consumption of fruits and vegetables and the consumption of unhealthy 'non-core' foods (i.e. snacks).

(2) To determine whether eating in response to external food cues is more prevalent in those with weak executive control as would be predicted by Temporal Self-Regulation Theory.

(3) To assess whether implementation intentions are more beneficial in those with weak executive control.

### Design

The role of executive control factors in dietary behaviours will be investigated in healthy young adults with known intentions about the consumption of (a) fruits and vegetables and (b) unhealthy snack foods. Performance on four executive tasks (each measuring a different component of executive control) will be used in a prospective design to predict the discrepancy between actual behaviour over three days and intended behaviour for the same time period. In addition, executive task scores will also be used to predict scores on the external eating subscale of the Dutch Eating Behaviour Questionnaire which measures eating in response to environmental cues [17], and used to compare the beneficial effect of implementation intentions in those with weak and strong executive control. Participants will complete executive tests and questionnaires in the laboratory setting and diary measures of their behaviour in the field.

It is hypothesised that scores on the executive tasks will predict a significant amount of the variance in the difference observed between actual and intended dietary behaviour, and specifically that those who score poorly on the executive tasks will show a larger discrepancy between intended and actual dietary behaviour than those who score well. In addition, it is expected that those with weak executive control will be more likely to eat in response to external food cues, and will benefit more than those with strong executive control from the formation of implementation intentions.

### Primary Outcomes

(1) Magnitude of the discrepancy between intended and actual consumption of (a) fruits and vegetables, and (b) unhealthy snack foods

(2) Score on the 'external eating' subscale of the Dutch Eating Behaviour Questionnaire (a measure of tendency to eat in response to food cues)

(3) Likelihood of performance of a simple pre-specified task (accessing a website and recording requested information) after the use of implementation intentions.

### Study Population

50 University undergraduate and postgraduate students at the University of Aberdeen will be sampled. This group will be used because students are typically young adults living away from home for the first time and as such are just beginning to be solely responsible for the food that they buy, cook and consume. It is at this point in life that many unhealthy dietary behaviours are likely to be established. In addition, as registered students, this group is likely to available for follow-up during their time at university.

### Inclusion and Exclusion Criteria

Participants must have English as their first language (to ensure comparability of performance on the language based executive tests).

### Recruitment

Participants will be primarily recruited through the University of Aberdeen School of Psychology's online research participation system – Sona Systems. In addition, posters and online advertising will be displayed across the University campus. Participants who are 1^st ^year psychology undergraduates will receive course credits for participation and participants who are not will be reimbursed at the recommended University participation rate of €5 per hour. Written informed consent will be obtained at the first scheduled appointment with each participant. Consent forms will summarise what is involved in the study, assure participants of their right to withdraw from the study at any time without penalty or having to give a reason, and provide an explanation of how and where the data they provide will be stored.

### Procedure & Materials

The study methodology has five distinct stages; (1) measurement of behavioural intentions and perceived control over behaviour, (2) completion of the executive control test battery, (3) completion of the Dutch Eating Behaviour Questionnaire, the Cognitive Failures Questionnaire, and the Dysexecutive Questionnaire, (4) completion of the 3-day behavioural diaries, and (5) the implementation intention task.

#### (1) Ratings of Intentions and Perceived Behavioural Control

Participants will come into the laboratory and fill out a self-report measure rating their intentions and perceived behavioural control on 6 different behaviours (eating fruits and vegetables, eating 'non-core' snack foods, studying/coursework, non-essential shopping, watching TV/DVDs, and exercising). Multiple behaviours are included so that participants are not alerted to the fact that the focus of the study is diet, as awareness may alter their report of food consumed. The target behaviours in this case are eating fruit and vegetables and eating unhealthy snack foods. Participants will be asked to quantify their intentions towards the behaviours of interest (i.e. to say how many portions of fruit and vegetables they intend to eat, and how much time they intend to spend studying etc) for each of the three days following the test session. In this way, the discrepancy between actual and intended behaviour can be calculated for later analysis. In addition to the intention ratings, participants will also be asked to estimate their perceived behavioural control (as a Theory of Planned Behaviour construct; [18] over each of the intended behaviours so that any effects of executive control can be distinguished from effects of perceived behavioural control.

#### (2) Executive Control Test Battery

Participants will complete four established psychological tests; The Go/NoGo (a replication of that used by [8]), the Tower test, a verbal fluency task and the Trail-Making test [19]. The Go/NoGo and Trail-Making task tap the two main identified components of executive control that are not highly related to intelligence (i.e. are purely executive) – inhibition of prepotent responses and mental set-shifting [20]. The verbal fluency test is used as measure of cognitive flexibility/shifting ability because flexible, creative patterns of thought may be associated with more successful behaviour change [21], and the Tower task as a measure of planning because setting out a plan in advance is know to improve the chances of successful behaviour change [10].

#### (3) Self-report questionnaires

Participants will be asked to complete the 33-item Dutch Eating Behaviour Questionnaire (DEBQ), as a measure of their typical eating style. The DEBQ is composed of 3 subscales – emotional eating, restrained eating and external eating and the latter provides an estimate of how much an individual's eating behaviour depends on external cues. This is of interest because Temporal Self-Regulation Theory [9] predicts that executive control and presence/absence of external cues in the environment will interact to alter the likelihood of behaviour occurring. So in theory, those with weak executive control should be more likely to eat in response to external cues. This study will provide some idea of whether the two are related and further planned studies will address this issue in more depth. Participants will also be asked to complete two self-report measures of executive control: the 20-item Dysexecutive Questionnaire, and the Cognitive Failures Questionnaire. These measures are included to see which aspects of executive control (as objectively measured) the self-report measures detect.

#### (4) 3-Day Behaviour Diaries

Participants will be given computerised diaries programmed to give an audible alarm 3 times a day for 3 consecutive days to prompt them to make a report of each of the intended behaviours. Using computerised diaries will ensure that diary entries are made when they are requested (rather than filled in retrospectively at the end of the measurement period) and multiple reports over the measurement period will reduce the time between behaviour and report which will reduce retrospective report bias. Upon hearing a diary alarm, participants will be asked to fill in how much of each behaviour of interest they have performed since the last diary alarm in a format that is comparable to that used for the intention items in Part 1. The diaries are Dell Axim X51s, and will run the questionnaire generation program Pocket Questionnaire [22].

#### (5) Implementation Intention Task

After return of the diaries, participants will be asked to complete the final task. They will be told that they should spend some time over the subsequent 24 hours thinking about their dietary behaviour and decide why they think they eat or drink things that are not in line with their intentions. Once they have come up with a few suggestions as to why this might happen in their particular case, they must log onto a website where they can report their findings. Each participant will be given the address of the website, and informed that the website is currently down but will be back online by the following day and they should complete the task at that time. One in three participants will be given no further instructions. The remaining two thirds will be asked to complete a short implementation intention task (i.e. to form an if-then plan) to help them to remember to complete the task and post their results on the website. This task asks participants to specify when and where they will complete and the task.

Finally, participants will be asked if they are willing to consent to being re-contacted one year later and asked to complete a short questionnaire on their dietary behaviour at that time. Debriefing for parts 1–4 of the experiment will occur at this point, and debriefing information for part 5 will be emailed to the participants after one week.

### Sample size

Sample size will be 50. This was determined using the GPOWER computer program for a-priori power analyses [23], for a multiple regression analysis with 4 predictor variables, alpha = 0.05, power = 0.90, and a large (0.35) effect of intention and executive function on change in consumption of fruit and vegetables. The effect size was estimated to be large in line with published findings [8].

### Ethical Approval/Peer review

This project has be subjected to full ethical review by the Psychology Ethics Committee (PEC) at the School of Psychology, University of Aberdeen, and approved. In addition the proposal has been through full peer review by the Scottish Government Chief Scientist Office.

### Analysis

#### Aim 1

The discrepancy between actual and intended behaviour on each of the two target behaviours will be calculated for the 3 days of the measurement period in total by subtracting the amount of actual behaviour from the amount of behaviour intended. This discrepancy score will be used as the dependent variable in a multiple linear regression model with the 4 executive control measures as predictor variables.

#### Aim 2

Total score on the external eating subscale of the Dutch Eating Behaviour Questionnaire will be used as the dependent variable in a multiple linear regression model with the 4 executive control measures as predictor variables to determine whether eating in response to external cues is more prevalent in those with weak executive control.

#### Aim 3

Those assigned to the implementation intentions condition in part 5 of the experiment will be divided (by median split) in terms of executive control scores into 'strong executive control' and 'weak executive control' groups. The rates of target behaviour (logging onto the website) in both groups will be compared to rates of behaviour in the control group using chi-square.

## Discussion

This work will build on published evidence of the role of executive control in implementing intended health behaviours by investigating which executive processes are most important for translating dietary intentions into action. If multiple executive processes are found to be involved in intention-behaviour translation then public health interventions could utilise this information by designing interventions aimed at reducing the demand on the identified executive processes.

If those with weak executive control are found to be more likely to eat in response to environmental cues, then this would have clear implications for advertising of unhealthy foods and the need for salient healthy eating messages in the public domain. Finally, if those with weak executive control benefit more than others from the formation of implementation intentions then this would provide evidence in favour of the use of implementation intentions within public health interventions.

## Competing interests

The author(s) declare that they have no competing interests.

## Authors' contributions

JA conceived of and designed the study in collaboration with MJ and NC. Data collection and analysis will be carried out by JA. This protocol was drafted by JA and revised/commented on by MJ and NC. All authors read and approved the final manuscript.

## Pre-publication history

The pre-publication history for this paper can be accessed here:


